# Cholecystokinin Outcome on Markers of Intestinal IgA Antibody Response

**DOI:** 10.3390/cimb44060173

**Published:** 2022-06-01

**Authors:** Juan Morales-Magaña, Ivonne Maciel Arciniega-Martínez, Maria Elisa Drago-Serrano, Aldo Arturo Reséndiz-Albor, Rosa Adriana Jarillo-Luna, Andrea Cruz-Baquero, Modesto Gómez-López, Fabiola Guzmán-Mejía, Judith Pacheco-Yépez

**Affiliations:** 1 Sección de Estudios de Posgrado e Investigación, Escuela Superior de Medicina del Instituto Politécnico Nacional, Plan de San Luis esq. Salvador Díaz Mirón s/n, Mexico City 11340, Mexico; jmoralesm1707@alumno.ipn.mx (J.M.-M.); rajarillo@ipn.mx (R.A.J.-L.); mgomezlo@ipn.mx (M.G.-L.); 2 Laboratorio de Inmunidad de Mucosas, Sección de Estudios de Posgrado e Investigación, Escuela Superior de Medicina del Instituto Politécnico Nacional, Plan de San Luis esq. Salvador Díaz Mirón s/n, Mexico City 11340, Mexico; iarciniega@ipn.mx (I.M.A.-M.); aresendiza@ipn.mx (A.A.R.-A.); 3 Departamento de Sistemas Biológicos, Universidad Autónoma Metropolitana, Unidad Xochimilco, Calzada del Hueso No. 1100, Mexico City 04960, Mexico; mdrago@correo.xoc.uam.mx (M.E.D.-S.); fabiolagm03@gmail.com (F.G.-M.); 4 Departamento de Formación Básica Disciplinaria, Escuela Superior de Medicina del Instituto Politécnico Nacional, Plan de San Luis esq. Salvador Díaz Mirón s/n, Mexico City 11340, Mexico; 5 Bacteriología, Facultad de Ciencias de la Salud, Universidad Colegio Mayor de Cundinamarca, Bogotá 111311, Colombia; candreacruz@unicolmayor.edu.co

**Keywords:** Cholecystokinin, devazepide, IgA^+^ plasma cells, TGF-β, CCKR, pIgR

## Abstract

Cholecystokinin 8 (CCK8) is an entero-octapeptide that participates in crosstalk with components of intestinal immunity via the CCK receptor (CCKR), but its role in modulation of the IgA response is not fully known under physiological conditions. Male eight-week-old BALB/c mice each were intraperitoneally injected once during 7 days with CCK8, devazapide (CCKR1 antagonist), L365,260 (CCKR2 antagonist) or vehicle (sham group). In intestinal lavages, total and secretory IgA (SIgA) were determined by ELISA; in lamina propria, IgA^+^ B lymphocytes and IgA^+^ plasma cells were analyzed by flow cytometry; mRNA levels of polymeric immunoglobulin receptor (pIgR) in epithelial cells and α chain, interleukins (ILs) in lamina propria cells were assessed by qRTPCR. Regarding the sham conditions, IgA^+^ plasma-cell percentage and IL-2, IL-5, IL-10 and transforming growth factor-β (TGF-β) mRNA levels were either increased by CCK8 or decreased by both CCKR antagonists. For IgA/SIgA responses, IL-4/IL-6 mRNA levels were decreased by all drugs and pIgR mRNA was increased by CCK8 and reduced by L365,260. IgA^+^ B cell percentage and α chain mRNA levels were elicited by CCK8 and L365,260. Data suggested a presumable differential role of CCK/CCKR on the IgA-response; outcome of L365,260 on the elicitation of IgA^+^ B cells and α chain mRNA needs further examination.

## 1. Introduction

Cholecystokinin (CCK) is an enteropeptide with both hormone and neurotransmitter activities secreted by endocrine I and L cells located predominantly in the small intestine [1,2]. Biologically active CCK comprises a COOH-terminal octapeptide (CCK8) with a tyrosine sulfated located in the seventh position from the COOH terminus and α-amidation of the COOH terminus [3]. CCK is secreted into the duodenum in response to the absorption of proteins and fats [4]. CCK activates intrinsic enteric-nerve fibers to convey information to extrinsic vagal afferent terminals innervating the lamina propria adjacent to the mucosal epithelium [5]. CCK also directly excites vagal afferent neurons that release acetylcholine (ACh) via the CCK1receptor (CCK1R) [5].

CCKRs comprise both CCK1R and CCK2R G protein-coupled receptors that send signals to increase the intracellular Ca^2+^ concentration and to activate protein kinase C [6,7]. CCK1R is expressed in the myenteric nerve plexus but not in the submucous nerve plexus of the antroduodenal region [8]. CCK2R is expressed neuronally in duodenal myenteric and vagal afferent nerve fibers and in nonneuronal cells such as mononuclear leukocytes and T lymphocytes [3]. Both CCK1R and CCK2R exist in high-, low- and very- low-affinity states. CCK1R displays higher affinity for CCK8 than CCK2R. The low- and very-low-affinity states of CCK1R predominate in an 80%. CCK1R is highly selective for sulfated analogues of CCK8 and for the antagonist devazepide (termed also L364,718) whereas CCK2R displays high affinity for both sulfated and nonsulfated peptide analogues of CCK8 and for the antagonist L365,260 [3]. Both devazepide and L365,260 are nonpeptidic CCKR antagonists, which is historically relevant since they were the first asperlicin derivatives retaining the 1,4-benzodiazepine skeleton. Devazepide is highly selective for CCK1R and displays potent activity by enteral and parenteral routes and even readily penetrates the blood–brain barrier; in humans, devazepide stimulates CCK-induced gallbladder contraction, gastric emptying and gastric motility; devazepide side effects include gallstone toxicity. L365,260 is a highly selective CCK2R antagonist; by the oral route, it inhibits gastrin-stimulated acid secretion efficiently in animal models. In humans, L365,260 displays a short-term and moderate activity on inhibition of gastrin-induced acid secretion and it is ineffective as anxiolytic; these findings seem to be related to its limited oral bioavailability by its low aqueous solubility [9].

Currently, CCK is regarded as an enteric neuroendocrine player with a role in the crosstalk with intestinal components of the immune response, such as immunoglobulin A (IgA), resulting in the maintenance of gut homeostasis [10]. Intestinal IgA response is determined by B cells expressing membrane monomeric IgA (mIgA^+^ B cells) primed at Peyer’s patches, a prominent organized inductive site of the gut-associated lymphoid tissue (GALT) [11]. After arriving at the intestinal lamina propria as the main intestinal effector site, immature mIgA^+^ B cells undergo clonal expansion and terminal maturation under the influence of interleukins to become mature plasma cells secreting dimeric (dIgA) or polymeric (pIgA) IgA [12]. Dimeric and polymeric IgA are taken up by polymeric immunoglobulin receptor (pIgR) expressed at the basolateral side of the epithelial gut monolayer; the protein complex is intracellularly transported via vesicle-mediated transcytosis, and until reaching the apical side, pIgR is cleaved to render a secretory component (SC) associated with dIgA and pIgA; the protein complex termed secretory IgA (SIgA) is released in the luminal milieu [13,14].

Experimental findings support the role of CCK in the control of IgA response. A study conducted in T-cell receptor α chain knockout mice showed that increased colonic inflammation was accompanied by a decreased number of IgA^+^ cells and CCK^+^ cells [15]. The beneficial outcome of CCK by promoting a gut increase in IgA levels was associated with the maintenance of B- and T-cell populations in the Peyer’s patches of the distal bowel, as documented in mice fed total parenteral nutrition [16]. In addition to acting as a potent inducer of gut antibody secretion, CCK also preserves gut health by attenuating both the IgA decrease and impaired mucosal immunity caused by stress glucocorticoid hormones [17,18].

Several experimental settings have shown that the modulatory effect of CCK on the intestinal IgA antibody response is blunted by CCKR antagonists, as documented in mice [19] and rats [17,20,21]. Moreover, CCK modulates Peyer’s patches and lamina propria B and T lymphocytes, and IgA-producing interleukins, as documented in mice [22,23,24].

Animal models that entail immunization, diet or drug interventions have evidenced the effect of CCK8 on IgA in terms of secretion/output [17,20,21,25] or in the context of intestinal immunity [15,16,19,22]. CCK8 impact on IgA response and the IgA-associated parameters of the intestinal immunity in animals without dietary, immunological or pharmacological manipulations, i.e., under physiological conditions, is unknown. This approach is worthwhile in order to gain more insights into the substantive role of CCK8 in intestinal mucosal immunity. Thus, this study aimed to perform for the first time an overall analysis of the impact of CCK8 and specific CCK1R and CCK2R antagonists on IgA antibody generation and IgA-associated interleukins in the small intestine of mice under physiological conditions; this included an assessment of pIgR involved in IgA transcytosis and intestinal homeostasis [13,14]. This study will provide an experimental foundation that will contribute to future insights into the role of CCK8 modulation in the intestinal IgA response with clinical impact on chronic dysfunctions such as inflammatory bowel diseases [26].

## 2. Materials and Methods

### 2.1. Animals

Eight-week-old male BALB/c mice (20–25 g) were obtained from our Animal Breeding Unit (Escuela Superior de Medicina, IPN) and were housed in plastic cages in groups of three. All mice were kept on a 12 h light:dark cycle (lights on at 6 a.m.) for two weeks prior to their experimental manipulation at 10 weeks of age. Food and water were provided ad libitum, and all manipulations and assays were conducted before 15:00 h to avoid any influence of the circadian cycles of adrenocorticotropin hormone (ACTH) and corticosterone. Animals were handled according to a protocol (ESM-CICUAL-04/13-01-2014) approved by the Institutional Animal Care and Use Committee in accordance with the ARRIVE guidelines for reporting animal research [27].

### 2.2. Drugs

Drugs were dissolved in sterile DMSO at the following dosage: CCK8 (8 μg/kg body weight, cat. no. C2901, Sigma, Saint Louis MO, USA) as a CCK agonist; devazepide (0.5 mg/kg, cat. no D3821, Sigma, Saint Louis MO, USA) as a CCK1R antagonist; and L365,260 (0.5 mg/kg, cat. no. sc-311370, Santa Cruz, TX, USA) as a CCK2R antagonist. Drugs were administered once/day for seven consecutive days by intraperitoneal injection. Thirty minutes after the last drug delivery on the 7th day, pentobarbital sodium salt (cat. no. P3761, Sigma, Darmstadt, Germany) at a lethal dose of 100 mg/kg body weight was administered intraperitoneally to proceed with euthanasia.

### 2.3. Experimental Protocol and Sampling

Groups with eight mice each were treated with CCK8, devazepide or L365,260; and a sham group treated with vehicle (sterile DMSO) was included. On the 7th day, 30 min after the last substance delivery, all mice were euthanized with pentobarbital sodium and exsanguinated by cardiac puncture. Thereafter, the entire small intestine was dissected and flushed with 5 mL of phosphate-buffered saline (PBS) containing 0.02% sodium azide and protease inhibitor cocktail (cat. no. 04693124001 complete mini, Roche Diagnostics, Mannheim, Germany). The washout material was centrifuged at 10,000× *g* for 20 min at 4 °C, and the supernatant corresponding to intestinal secretions was stored at −70 °C until the measurement of total IgA and SIgA by ELISA. After washing, small-intestine tissue samples from each mouse were collected to prepare single-cell suspensions of epithelial cells and lamina propria. Epithelial cells were isolated for detecting polymeric immunoglobulin receptor (pIgR); in purified lamina propria cells, mRNA levels of α chain, interleukins (IL-2, IL-4, -IL-5, IL-6, IL-10) and TGF-β were determined by qRT-PCR; lamina propria cell suspensions were prepared for assessing IgA^+^ B lymphocytes and IgA^+^ plasma cells by flow cytometry.

### 2.4. Enzyme-Linked Immunosorbent Assay

Total IgA and SIgA in intestinal secretions were determined on base an ELISA sandwich protocol as follows: 96-well ELISA plates were coated for total IgA with goat antimouse IgA antibody (cat. no. 626700 Thermo Fisher Scientific, Rockford, IL, USA) and for SIgA with goat anti-SC antibody (C-2) (cat. no. sc-374343 Santa Cruz, CA, USA) in carbonate–bicarbonate buffer pH 9.6 and incubated overnight incubated at 4 °C. Six rounds of washing with 0.05% Tween 20 in phosphate-buffered saline (PBS-T) were performed before and after blocking the nonspecific binding sites with 6% nonfat milk in PBS-T for 2 h at 37 °C. Nex, the microplates were treated with intestinal liquid samples diluted in PBS-T, tested in triplicate and incubated overnight at 4 °C. Six rounds of washing with PBS-T were applied before and after the microplate incubation with goat antimouse IgA horseradish peroxidase conjugate in PBS-T for 2 h at 37 °C (cat. no. 626720 Thermo Fisher Scientific, Rockford, IL, USA). Then, the microplates were treated with the substrate containing ortophenylendiamine (cat. no. AC13055250 Thermo Scientific, Philadelphia, PA, USA) plus hydrogen peroxide in citrate–phosphate buffer pH 5.0 for 15 min at room temperature in the dark. The enzymatic reaction was arrested with 2.5 M sulfuric acid before reading the absorbance at 492 nm. The concentration of total IgA (μg/mL) was quantified based on a standard curve of purified mouse IgA κ chains (cat. no. M1421 Sigma, Saint Louis, MO, USA) and the levels of SIgA expressed as relative absorbance values computed with a pool of intestinal liquids were expressed as the mean and standard deviation (SD).

### 2.5. Isolation of Lamina Propria Lymphocytes

The isolation of intestinal lymphocytes was carried out as previously described [26]. Briefly, the small intestine was removed, carefully cleaned from its mesentery and flushed of fecal contents with cold RPMI medium. Peyer’s patches were carefully removed from the small intestine before processing. Then, the small intestinal segments were everted by introducing an iron crochet needle tied to a string. The everted small intestine was transferred to a 50 mL tube containing 25 mL RPMI medium with 60 U/mL of type IV collagenase (Sigma), 1% FCS and 50 μg/mL gentamicin. The tubes were incubated horizontally for 30 min at 37 °C in a shaking-water bath at 180 rpm. The contents of each tube were then transferred to Petri dishes and 200 mL FCS was added. The intestinal mucosa was compressed with a syringe plunger over a plastic mesh, single-cell suspensions containing lamina propria cells were filtered through organdy mesh and then centrifuged for 10 min at 2500× *g*. Cell suspensions were collected and centrifuged in a discontinuous 40%/70% Percoll gradient at 600× *g* for 30 min. Cells from the interface were washed and suspended in RPMI medium.

### 2.6. Flow-Cytometry Assays

The isolation of lamina propria cells from the small intestine was conducted as described previously, and the cell suspensions were adjusted to 1 × 10^6^ cells/mL in PBS [28]. The surface phenotypes of B cells were detected by using labeled monoclonal anti-CD45/B220/PerCP, anti-CD19 PE, and anti-IgA FITC (all from BD Biosciences). The cells were incubated for 25 min at room temperature. Finally, the cells were washed with PBS and fixed in paraformaldehyde at 1%. The plasma cells were labeled with anti-CD19 PE and anti-CD138 APC antibodies (all from BD Biosciences, San Jose, CA, USA). For intracellular IgA^+^ detection, the cells were fixed, permeabilized and stained with anti-IgA FITC according to the BD Bioscience protocol for intracellular staining [29,30,31,32]. The fluorescence signal intensity was recorded and analyzed by a FACSAria flow cytometer (Becton Dickinson Biosciences San Jose, CA, USA). Events were collected from the lymphocyte gate on the FSC/SSC dot plot. A total of 20,000 gated events were acquired from each sample using BD FACSDiva^TM^ software 6.1 (Becton Dickinson Biosciences, San Jose, CA, USA). Data for each group are expressed as the mean ± standard deviation (SD), and the comparisons were analyzed using Summit software v4.3 (Agilent Dako, Fort Collins, CO, USA).

### 2.7. Epithelial-Cell Isolation

To isolate the epithelial cells, the small intestine was incubated in RPMI 1640 with 1 mM dithiothreitol (Sigma) and 1.5 mM EDTA (Sigma), with shaking at 150 rpm for 30 min at 37 °C. The cell suspension obtained was passed through organza to remove the mucus and centrifuged at 1811× *g* for 10 min at 4 °C. The pellet was suspended in 15 mL of RPMI 1640, passed through organza, and washed two times in 15 mL of RPMI 1640 by centrifugation at 1811× *g* for 10 min at 4 °C. The washed pellet was suspended in 20% Percoll and centrifuged over a discontinuous Percoll gradient at 515× *g* for 30 min at 25 °C. Epithelial cells were recovered at the interphase between 20% and 44%, then washed in PBS and centrifuged as indicated above. The cell preparation was suspended in RPMI 1640. The purity of the samples was analyzed by light microscopy on the basis of the normal morphology for epithelial cells. Samples contained up to 85% epithelial cells. Viability, determined by trypan blue exclusion, was 90% [26].

### 2.8. RT-qPCR Assays

The mRNA expression of pIgR was analyzed in epithelial cells, whereas mRNA expression of IgA α chain and IL-2, IL-4, IL-5, IL-6, IL-10 and TGF-β were analyzed in lamina propria cells. Epithelial cells and lamina propria cells were isolated according to the previous protocol [26,28,29]. Quantitative real-time polymerase chain reaction (qRT-PCR) was performed. In brief, total mRNA was extracted using TRIzol reagent (50 mg/mL) according to the manufacturer’s protocol (cat. no. 15596018, Invitrogen, CA, USA). Reverse transcription was performed with a cDNA synthesis kit (cat. no. 28025021 Invitrogen) according to the manufacturer’s instructions. cDNA amplification by PCR was performed with a LightCycler TaqMan Master reaction mix (cat. no. 04535286001 Roche), LightCycler capillaries (cat. no. 11909339001 Roche) according to the manufacturer’s instructions. Amplification in a LightCycler Carousel-Based System 1.5 Instrument (cat. no. 04 484 495 001 Roche) was conducted as follows: 10 min at 95 °C, followed by 50 cycles of 10 s at 94 °C, 20 s at 90 °C and 5 s at 72 °C. Original specific oligonucleotide primers were generated by using the online assay-design software ProbeFinder (https://lifescience.roche.com/en_mx/brands/universal-probe-library.html (accessed on 17 July 2018) as previously reported (30). The nucleotide sequences of the primers used for RT-qPCR are shown in Table 1. Universal ProbeLibrary probes were from Roche Diagnostics. The samples were analyzed in duplicate, and the mRNA expression levels were calculated by using the comparative parameter-quantification-cycle method and normalized to the level of the 18S ribosomal RNA subunit [33].

### 2.9. Statistical Analysis

Data are presented as the mean and standard deviation (SD) of at least 3 independent assays (*n* = 8 mice per group). Comparisons among three independent groups were analyzed by using one-way ANOVA. All analyses were performed with the statistical program SigmaPlot for Windows version 11 (Systat Software Incorporated, CA, USA) and differences among the groups were analyzed with post hoc Tukey test. Significant differences are expressed as * *p* < 0.05, ** *p* < 0.01, *** *p* < 0.001 or **** *p* < 0.0001.

## 3. Results

### 3.1. Total IgA and SIgA Antibody Responses

**Figure 1:**
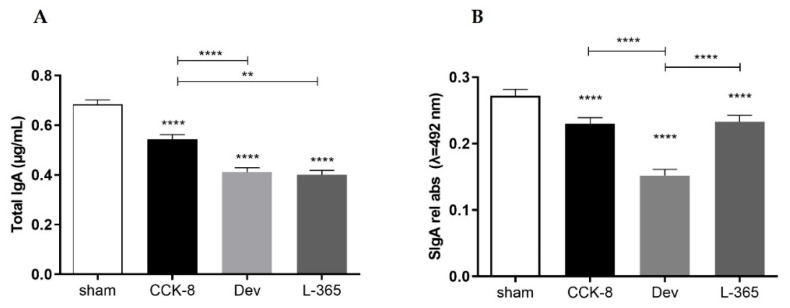
Total IgA (μg/mL) (**A**) and SIgA (relative absorbance λ = 492 nm) (**B**) in intestinal secretions from the small intestine were assessed in the following groups: sham, CCK8, devazepide and L365,260. The results from one representative experiment (from three replicates) with eight mice per group are expressed as the mean and standard deviation (SD). ** *p* < 0.01 and **** *p* < 0.0001 upon column comparisons vs. control group or upon line comparisons among the CCK8, devazepide and L365,260 groups.

Assessment of intestinal antibody responses indicated that relative to the sham group, the total IgA concentration was significantly reduced in the CCK8, devazepide and L365,260 groups (*p* < 0.0001, Figure 1A); within the treated groups, the total IgA concentration was significantly higher in the CCK8 than in the devazepide (*p* < 0.0001) and L365,260 (*p* < 0.01) groups.

Compared with the sham group, the SIgA response was found also significantly decreased in the CCK8, devazepide and L365,260 groups (*p* < 0.0001, Figure 1B); within the treated groups, the SIgA levels in the CCK8 and L365,260 groups were higher than in the devazepide group (*p* < 0.0001).

### 3.2. Percentages of IgA^+^ B Lymphocytes and IgA^+^ Plasma Cells

**Figure 2:**
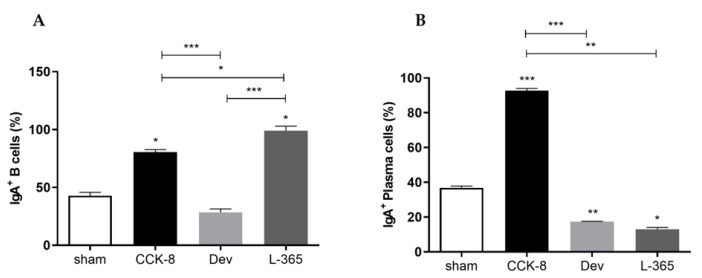
Percentage (%) of IgA^+^ B lymphocytes (**A**) and IgA^+^ plasma cells (**B**) in the lamina propria of the sham-, CCK8-, devazepide- and L365,260-treated groups. The results from one representative experiment (from three replicates) with eight mice per group are expressed as the mean and standard deviation (SD). * *p* < 0.05, ** *p* < 0.01 *** *p* < 0.001 and upon column comparisons vs. control group or upon line comparisons among the CCK8, devazepide and L365,260 groups.

By comparison with the sham group, the percentage of IgA^+^ B lymphocytes was significantly higher in the CCK8 and L365,260 groups (*p* < 0.05, Figure 2A); within the treated groups, the percentage of IgA^+^ B lymphocytes was significantly higher in the L365,260 group than in the CCK8 (*p* < 0.05) and devazepide (*p* < 0.001) groups and higher in the CCK8 group than in the devazepide group (*p* < 0.001, Figure 2A).

In comparison with the sham group, the percentage of IgA^+^ plasma cells was significantly higher in the CCK8 (*p* < 0.001) group or lower in both the devazepide (*p* < 0.01) and L365,260 (*p* < 0.05) groups. Within the treated groups, the percentage of IgA^+^ plasma cells was higher in the CCK8 group than in the devazepide (*p* < 0.001) and L365,260 groups (*p* < 0.01, Figure 2B).

### 3.3. mRNA Expression of α chain and pIgR

**Figure 3:**
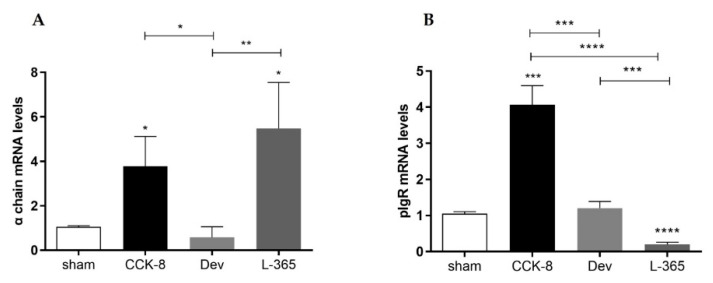
Relative mRNA expression of α chains in lamina propria cells (**A**) and pIgR in epithelial cells isolated from the small intestine (**B**) of sham-, CCK8-, devazepide- and L365,260-treated mouse groups. The results from one representative experiment (from three replicates) with eight mice per group are expressed as the mean and standard deviation (SD). * *p* < 0.05, ** *p* < 0.01 *** *p* < 0.001 and **** *p* < 0.0001 upon column comparisons vs. control group or upon line comparisons among the CCK8, devazepide and L365,260 groups.

Compared with the sham group, the α chain mRNA expression was higher in the CCK8 and L365,260 groups (*p* < 0.05 Figure 3A). Within the treated groups, α chain mRNA expression was higher in the CCK8 (*p* < 0.05) and L365,260 (*p* < 0.01) groups than in the devazepide group (Figure 3A). Relative to the sham group, pIgR mRNA levels were higher in the CCK8 group (*p* < 0.001) or lower in the L365,260 group (*p* < 0.0001); moreover, pIgR mRNA expression was higher in the CCK8 group than in the devazepide (0.001) and L365,260 groups (*p* < 0.0001) and higher in the devazepide group than in the L365,260 group (*p* < 0.001, Figure 3B).

### 3.4. Relative Expression of Interleukins and TGF-β in Lamina Propria Cells

**Figure 4:**
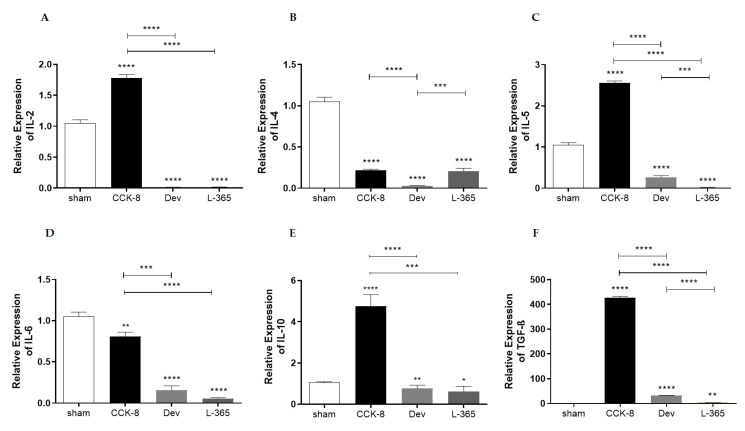
Relative expression of interleukins (ILs) in lamina propria cells of the following groups: sham, CCK8, devazepide, and L365,260. (**A**) IL-2, (**B**) IL-4, (**C**) IL-5, (**D**) IL-6, (**E**) IL-10 and (**F**) transforming growth factor-β (TGF-β). The results from one representative experiment (from three replicates) with eight mice per group are expressed as the mean and standard deviation (SD). * *p* < 0.05, ** *p* < 0.01 *** *p* < 0.001 and **** *p* < 0.0001 upon column comparisons vs. control group or upon line comparisons among the CCK8, devazepide and L365,260 groups.

Relative-expression assays were performed to analyze the expression of IgA-associated interleukins and TGF-β in lamina propria cells. Compared with those in the lamina propria of the sham group, IL-2 mRNA levels were higher in the CCK8 group or lower in the devazepide and L365,260 groups (*p* < 0.0001). Within the treated groups IL-2 mRNA levels were significantly higher in the CCK8 than in the devazapide and L365,260 groups (*p* < 0.0001, Figure 4A).

Analysis of IL-4 mRNA levels showed that in comparison with the sham group, IL-4 mRNA levels were lower in the CCK8, devazepide and L365,260 groups (*p* < 0.0001); within the treated groups, IL-4 mRNA levels were higher in the CCK8 (*p* < 0.0001) and L365,260 groups (*p* < 0.001) than in the devazepide group (Figure 4B).

Compared with the sham group, the IL-5 mRNA levels were higher in CCK8 or lower in the devazepide and L365,260 groups (*p* < 0.0001). Within the treated groups, IL-5 mRNA levels were significantly higher in the CCK8 than in the devazepide and L365,260 groups (*p* < 0.001) and higher in the devazepide than in the L365,260 group (*p* < 0.001 Figure 4C).

Analysis of IL-6 mRNA levels showed that in comparison with the sham group, IL-6 mRNA levels were lower in the CCK8 (*p* < 0.01), devazepide and L365,260 groups (both *p* < 0.0001, Figure 4D); within the treated groups, IL-6 mRNA levels were higher in the CCK8 than devazepide (*p* < 0.001) and L365,260 (*p* < 0. 0001) groups (Figure 4D).

Analysis of IL-10 mRNA levels indicated that in regard to the sham group, IL-10 mRNA levels were higher in the CCK8 group (*p* < 0.0001) or lower in the devazepide (*p* < 0.01) and L365,260 groups (*p* < 0.05); within the treated groups, IL-10 mRNA levels were higher in the CCK8 than in the devazepide (*p* < 0.0001) and L365,260 groups (*p* < 0.001, Figure 4E).

Compared with those of the sham group, the TGF-β mRNA levels were higher in the CCK8 and lower in the devazepide (*p* < 0.0001) and L365,260 groups (*p* < 0.01, Figure 4F). Within the treated groups, TGF-β mRNA levels were higher in the CCK8 group than in the devazepide and L365,260 groups (both *p* < 0.0001) and greater in the devazepide group than in the L365,260 group (*p* < 0.0001, Figure 4F).

## 4. Discussion

In animal models that entail immunization, diet or drug interventions, the effect of CCK8 on IgA has been in terms of output [17,20,21,25] or in the context of intestinal immunity [15,16,19,22,25]. This study investigated for the first time the effects of CCK8 and CCK1R and CCK2R antagonists on selected parameters associated with the intestinal IgA antibody response in mice under physiological conditions.

An overall data analysis evidenced that regarding sham conditions, total IgA and SIgA responses were downmodulated by all drugs. As far as we know, the CCK2R effect on IgA output has not yet been documented, whereas CCK1R regulation on the decrease in IgA secretion at the intestinal level has been reported with devazepide [19] and with other CCK1R antagonists, proglumide [20] and loxiglumide (also termed CR1505) [21]. Previous assays have documented the effect of CCK8 on the upmodulation of the IgA antibody levels in gut secretions from animals fed total parenteral nutrition [16], treated via intraduodenal with oleic acid [21] or immunized intraperitoneally with ovoalbumin [20]. Unlike the aforementioned findings, in current contribution the levels of IgA were decreased by CCK8, and these apparent discrepancies may ascribe to the protocol of study conducted in mice provided free access to food and water. The outcome of a decrease in IgA by all drugs needs further assessment.

Data analysis indicated that in comparison with sham conditions, the percentage of IgA^+^ B cells from lamina propria and α chain mRNA expression displayed a similar pattern, i.e., upmodulation by the CCK8 agonist and CCK2R antagonist L365,260 and no significant changes with CCK1R antagonist devazepide. In culture assays of murine Peyer’s patches, CCK8 did not enhance B-cell proliferation [22]. Thus, findings suggested that effects of CCK8 on IgA^+^ B cell growing may depend on the cell source, i.e., Peyer’s patches are enriched in immature IgA^+^ B lymphocytes with a high rate of proliferation or lamina propria as a prominent gut effector site where differentiation and maturation of IgA^+^ B cells toward terminally differentiated and nonproliferating IgA^+^ plasma cells take place [11]. Data may also suggest that IgA^+^ B lymphocytes exhibit a divergent pattern of expression and/or affinity of CCK2R according to the cell source. Although IgA^+^ B lymphocyte proliferation may result from signals mediated by the ligation between CCK2R and L365,260, other IgA^+^ B-cell interactions via receptors with factors that favored their clonal expansion secreted in the gut milieu by immune cells may be involved; to our knowledge, assessment of CCK1R and CCK2R in IgA^+^ B cells at intestinal milieu has not been reported, but this aspect has been documented in splenic B cells expressing both CCK1R and CCK2R [34].

Regarding the sham conditions, the IgA^+^ plasma-cell percentage was elicited by the CCK8 agonist or reduced by both CCKR antagonists. Thus, CCKR modulation on the differential response of immature IgA^+^ B cells and mature IgA^+^ plasma cells underlie the relevance of the extent of cell maturation. Data may reflect the interplay of CCKRs and T-cell receptor signals resulting in the activation of T-helper lymphocytes and the concomitant release of soluble factors associated to IgA generation; these include (TGF-β), essential for IgM^+^ to IgA^+^ class switch on B cells at Peyer’s patches; and interleukins that favored the clonal proliferation, maturation and terminal differentiation of IgA^+^ B cells to IgA^+^ plasma cells in gut lamina propria [12].

In this study, pIgR mRNA expression was triggered by the CCK8 agonist, inhibited by the CCK2R antagonist L365,260 or unaltered by the CCK1R antagonist devazepide. Data suggested a modulatory role of CCK2R on pIgR mRNA expression. The latter assumption is based on findings raised from molecular profiling in gastric mucosa of mice, which indicated that agonists with high affinity for CCK2R, i.e., CCK and its gastric analog, gastrin, modulate the pIgR mRNA expression and interferon γ (IFNγ) seem to be involved [35]. The underlying mechanism accounting for the pIgR mRNA decrease by L365,260 via CCK2R may be attributed to the downregulation of inflammatory cytokines that promote pIgR gene expression, including IFNγ, IL-1 and tumor necrosis factorα (TNFα). Moreover, IL-4 via the IL-4 membrane receptor triggers the activation of the transducer and activator of transcription (STAT6), which is targeted to the nucleus to synergize the effect TNFα on the upmodulation of pIgR gene expression; the TNFα signal pathway drives the activation of the nuclear factor κB (NFκB) families of transcriptional factors that trigger the pIgR expression [13]. Although pIgR mRNA levels were increased by CCK8 or decreased by L365,260, future assays are necessary to confirm whether the decreased IgA antibodies are related with a defective pIgR protein expression. As described previously, IgA-transcytosis via pIgR seems to involve both enteroendocrine-derived CCK and neurotransmitters released by cholinergic nerves [20]. The involvement of CCK8 in IgA transcytosis via pIgR is unclear; however, CCK8-induced elicitation of IgA seems to be under the control of luminal voltage-dependent ion calcium channels (inhibited with verapamil) and by the contraluminal Na^1+^, K^1+^, and 2Cl^1-^ cotransporters (inhibited by furosemide) [18]. Transcytosis and luminal release of IgA via pIgR is triggered by increased levels of intracellular ion calcium Ca^2+^ through the phospholipase C (PLC), phosphatidylinositol 4,5 bisphosphate (PI 4,5P2) and inositol 1,4,5 triphosphate (IP3) signaling pathways [36].

In the current study, CCK8 triggered while CCKRs antagonists decreased the relative mRNA expression of IL-2, IL-5, IL-10 and TGF-β in lamina propria cells. IL-2 acts as a potent T-cell factor for clonal expansion. IL-5 and IL-10 collaborate in the homing of IgA^+^ B cell from Peyer’s patches to the lamina propria, their clonal expansion and their maturation to become terminally differentiated IgA^+^ plasma cells, whereas TGF-β determines the IgM to IgA class switch in B cells [12]. Data suggest an interplay of CCKRs and T-cell receptor signals resulting in the transcription of ILs with a key role in IgA^+^ B cell generation that may mirror the findings on IgA^+^ plasma-cell response described before.

Regarding the sham conditions, the relative mRNA expression of IL-4 and IL-6 in lamina propria cells was found to be reduced in CCK8 and CCKR antagonist-treated groups. Molecular mapping indicated that both CCKR1 and CCKR2 share signaling pathways to trigger NFκB, AP1, STAT3 among some others [7,37]. As documented in vitro cultures of spleen cells, CCK8 did not alter the protein production of both IL-4 and IL-6 [34] but increased the IL-4 mRNA expression on T cells [38]. Data suggested the CCK8 signal via CCKRs resulting in IL-4 and IL-6 mRNA expression may depend on the cell source of B lymphocytes.

As conclusion, our data suggested that CCK8 favored the (i) clonal expansion, differentiation of IgA^+^ B cells as well as the IgA-associated IL-2, IL-5, IL-10 and TGF-β resulting in the increase in IgA^+^ plasma cells (ii) CCK8 increased the pIgR and α chain mRNA expression although this outcome was not reflected in IgA/SIgA antibody levels. CCK2R apparently modulated pIgR mRNA in epithelial cells; moreover, upmodulatory effects of L365,260 on IgA^+^ B cells and α chain mRNA suggested that in lamina propria it acts as CCK2R agonist, although this aspect has not been documented previously; the modulatory impact of CCK1R was evidenced in IgA^+^ plasma cells without affecting IgA^+^ B cells and α chain mRNA levels. Future assays are needed to address the substantive role of both CCKRs on the control of total IgA and SIgA responses and IL-4 and IL-6 mRNA expression.

This study provides an experimental foundation that will contribute to future insights into the role of CCK8 modulation in the intestinal IgA response with clinical impact on chronic dysfunctions such as inflammatory bowel diseases [26].

## Figures and Tables

**Table 1:** mRNA expression of genes.

Gene	ID	Forward Primer 5′-3′	Reverse Primer 5′-3′
α chain	1030660614	cgtccaagaattggatgtga	agtgacaggctgggatgg
pIgR	188247439	agtaaccgaggcctgtcctt	gtcactcggcaactcagga
IL-2	144305B06	gctgttgatggacctacagga	ttcaattcttgtggcctgctt
IL-4	1030660620	catcggcattttgaacgag	acgtttggcacatccatctc
IL-5	188247437	acattgaccgccaaaaagag	atccaggaactgcctcgtc
IL-6	1030660602	actaccaaactggatataatcagga	ccaggtagctatggtactccagaa
IL-10	1030660622	cagagccacatgctcctaga	tgtccagctggtcctttgtt
TGF-β	188247430	tggagcaacatgtggaactc	gtcagcagccggttacca

## Data Availability

Full data are contained in the present manuscript.
